# Branching morphogenesis of the urinary collecting system in the human embryonic metanephros

**DOI:** 10.1371/journal.pone.0203623

**Published:** 2018-09-07

**Authors:** Hana Ishiyama, Aoi Ishikawa, Haruka Kitazawa, Sena Fujii, Jun Matsubayashi, Shigehito Yamada, Tetsuya Takakuwa

**Affiliations:** 1 Human Health Science, Graduate School of Medicine, Kyoto University, Kyoto, Japan; 2 Congenital Anomaly Research Center, Graduate School of Medicine, Kyoto University, Kyoto, Japan; Leibniz Institute on aging - Fritz Lipmann Institute (FLI), GERMANY

## Abstract

An elaborate system of ducts collects urine from all nephrons, and this structure is known as the urinary collecting system (UCS). This study focused on how the UCS is formed during human embryogenesis. Fifty human embryos between the Carnegie stage (CS) 14 and CS23 were selected from the Kyoto Collection at the Congenital Anomaly Research Center of Kyoto University, Japan. Metanephroses, including the UCS, were segmented on serial digital virtual histological sections. Three-dimensional images were computationally reconstructed for morphological and quantitative analyses. A CS timeline was plotted. It consisted of the 3-D structural morphogenesis of UCS and quantification of the total amount of end-branching, average and maximum numbers of generations, deviation in the metanephros, differentiation of the urothelial epithelium in the renal pelvis, and timing of the rapid expansion of the renal pelvis. The first UCS branching generation occurred by CS16. The average branching generation reached a maximum of 8.74 ± 1.60 and was already the twelfth in CS23. The total end-branching number squared between the start and the end of the embryonic period. UCS would reach the fifteenth branching generation soon after CS23. The number of nephrons per UCS end-branch was low (0.21 ± 0.14 at CS19, 1.34 ± 0.49 at CS23), indicating that the bifid branching occurred rapidly and that the formation of nephrons followed after. The renal pelvis expanded mainly in CS23, which was earlier than that reported in a previous study. The number of nephrons connected to the UCS in the expanded group (246.0 ± 13.2) was significantly larger than that of the pre-expanded group (130.8 ± 80.1) (*P* < 0.05). The urothelial epithelium differentiated from the zeroth to the third generations at CS23. Differentiation may have continued up until the tenth generation to allow for renal pelvis expansion. The branching speed was not uniform. There were significantly more branching generations in the polar- than in the interpolar regions (*P* < 0.05). Branching speed reflects the growth orientation required to form the metanephros. Further study will be necessary to understand the renal pelvis expansion mechanism in CS23. Our CS-based timeline enabled us to map UCS formation and predict functional renal capacity after differentiation and growth.

## Introduction

Adult human kidneys contain approximately one million nephrons. The exact number of nephrons varies with each individual and determines innate functional renal capacity, as these units prepare glomerular filtrate [1,2]. The urinary collecting system (UCS) comprises a series of elaborate ducts that collect urine from all nephrons and transfer it to the bladder via the urothelial duct.

The UCS is a highly organized and regulated structure [3–10]. The proximal UCS consists of the renal pelvis and the calyx from which the collecting ducts are distributed. These ducts are formed by bifid branching during the embryonic and fetal periods where bifurcation occurs approximately fifteen times. Normal bifid branching is followed by arcade formation and nephron unit connection [3–6]. The UCS remodels following a highly regulated pattern while the nephrons are connected to the thirteenth to fifteenth generations of bifurcated branching during later development stages [6,11,12].

The proximal UCS is elastic and can alter its diameter according to the instantaneous volume of urine being produced, which changes according to physiological conditions. Hypertension and stagnation in the UCS adversely affect the urine production system. They can cause hydronephrosis, retrograde infection, and a loss of kidney function [13]. The (transitional) urothelial epithelium is distributed through the urinary tract, renal pelvis and calyx, urinary ducts, and bladder [14,15]. It is highly elastic and in classical studies on the human metanephros [6, 11,16], the fusion of bifurcated branches was proposed as the mechanism underlying proximal UCS expansion. The first 3–5 generations of tubules produced by ampullary division dilate to form the renal pelvis. The next 3–5 generations dilate to form the minor calyces, the papillae, and the cribriform plates. The next 6–9 generations develop into the collecting ducts [11,16].

Several studies have investigated and reported on UCS development [6,11,12,16]. Before Carnegie Stage (CS), crown-rump length (CRL) was used as a metric for UCS development [17,18]. CRL is simply a sample measurement including the standard deviation but does not completely reflect developmental stages. O’Rahilly and Muecke [19] stressed the importance of using staged human embryos to determine the precise course of developmental events.

The present study focused on how the UCS develops during the human embryonic period. We reconstructed a 3-D UCS branching tree based on serial histological sections and analyzed them both morphologically and morphometrically. We observed the urothelium-covered epithelium of the zeroth to third UCS generations. We also plotted a precise timeline based on Carnegie stages, and demonstrated that the renal pelvis rapidly expands within a narrow timeframe.

## Materials and methods

### Human embryo specimens

Approximately 44,000 human embryos, comprising the Kyoto Collection, are stored at the Congenital Anomaly Research Center of Kyoto University [20,21]. In most cases, pregnancies were terminated during the first trimester for socioeconomic reasons under the Maternity Protection Law of Japan. The samples were collected from 1963 to 1995 according to the regulations pertaining to each time period. Aborted embryos brought to the laboratory were measured, examined, and staged using the criteria of O’Rahilly and Müller [17]. Fifty human embryos between CS14 and CS23 were selected from the Kyoto Collection (CS14, n = 4; CS15, n = 5; CS16, n = 5; CS17, n = 5; CS18, n = 5; CS19, n = 5; CS20, n = 5; CS21, n = 5; CS22, n = 6; CS23, n = 5). All samples were free of overt damage and anomalies. Whole embryonic samples were fixed with 10% formalin, embedded in paraffin, and serially sectioned to a thickness of 10 μm. These samples were stained with hematoxylin-eosin, and histological specimens were properly preserved.

### Digitalization of the histological sections and 3-D reconstruction of the metanephros

Serial transverse sections (10 μm thickness) of whole embryos were digitalized with an Olympus virtual slide system (VS120-S5-J; Olympus Corp., Tokyo, Japan) for histological observations and 3-D reconstructions [22]. Sequential 2-D images at 25× magnification were digitally cropped around the metanephros. The metanephros, including the UCS, was segmented into serial digital sections. Three-dimensional images were computationally reconstructed and their morphology was analyzed using Amira v. 5.5.0 (Visage Imaging GmbH, Berlin, Germany). The total number of nephrons and those connected to the UCS were counted.

### Morphometric analysis and generation of the tree of UCS

The longitudinal metanephros length (mm) and the volumes of the UCS and the whole metanephros (mm^3^) were calculated with Amira v. 5.5.0 (Visage Imaging GmbH, Berlin, Germany). The UCS volume was that of the segment between the zeroth- and fifth metanephros branching generations. The 3-D coordinates of all branching points were acquired using Amira 5.5.0 (Visage Imaging GmbH, Berlin, Germany). The coordinates were analyzed with Matlab v. R2017b (MathWorks, Inc., Natick, MA, USA) to calculate the generation of all branches. The following parameters were calculated according to each CS: (a) average UCS end-branching generation; (b) total amount of UCS end-branching; and (c) maximum UCS end-branching generation. The four lobes were defined as upper-polar, upper-interpolar, lower-interpolar, and lower-interpolar according to the second UCS branching generation. The polar lobe (PL) group included the upper- and lower-polar lobes whereas the interpolar lobe (IL) group included the upper- and lower-interpolar lobes. Deviations of the end-branching between the PL- and IL groups were statistically analyzed.

### Statistics

The Wilcoxon signed-rank test was used to determine the deviation of the distributions among the four lobes. The Mann-Whitney *U* test was used to compare each parameter between the pre-expanded- and expanded groups and between the PL- and IL groups. These tests were run on SPSS v. 22 (IBM Corp., Armonk, NY, USA). The ethics committee of the Kyoto University Graduate School and Faculty of Medicine approved this study (E986, R0316).

## Results

### Morphology and morphometry of the metanephros and urothelial branching system during CS14 and CS23

**Fig 1:**
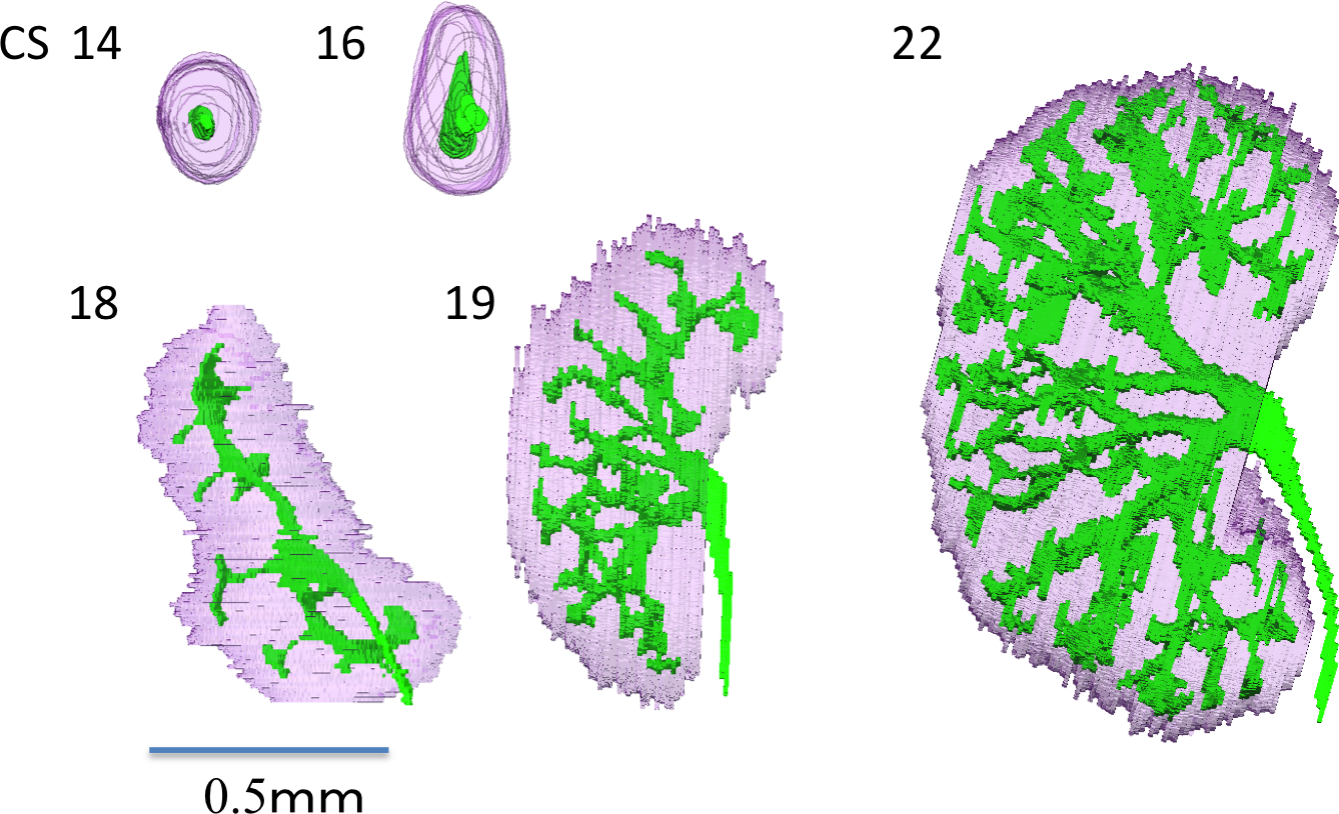
Morphogenesis of the urothelial branching system during CS14 and CS22. Development of the metanephroses and urinary collecting system during embryonic periods. Numbers indicate Carnegie stage. Scale bar = 0.5 mm.

Ureteric budding from the mesonephric duct and metanephric blastema first appeared in CS14 (Fig 1; S1–S11 Videos). The ureteric bud elongated cranially at CS14 then caudally at CS15. The metanephros was spherical and smooth at CS15 and it elongated along the craniocaudal axis at CS16. The longitudinal length, UCS, whole metanephros volume, and UCS branching generations of the metanephros increased. Its longitudinal length was 0.27 ± 0.02 mm at CS14 and 1.27 ± 0.20 mm at CS22. Therefore, there was a 1-mm increase between CS14 and CS22. The longitudinal length increased rapidly between CS22 and CS23, reaching 1.77 ± 0.23 mm at CS23. Therefore, it increased by 0.5 mm in one stage. Kidney volume and UCS also significantly increased between CS22 and CS23. According to the standard deviations, the variations in parameters such as the volumes of the UCS and kidney and the total amount of UCS end branching were extremely high at CS23.

UCS branching morphogenesis started at CS15. For all ten samples, the first branching generation occurred by CS16 (Table 1). Both the maximum and average numbers of UCS end-branchings increased with CS. The average branching generations were 1.62 ± 0.36 at CS17 and 3.05 ± 0.83 at CS18. The first and second branching generations elongated almost in two dimensions. As branching proceeded after the third generation, the tree became three-dimensionally distributed. Therefore, the metanephros thickened dorsoventrally and had a ‘bean’ shape after CS19. The average branching generation reached a maximum of 8.74 ± 1.60 at CS 23. The maximum branching generations were 1.9 ± 0.6 in CS17 and 6.5 ± 0.7 in CS19. Therefore, branching increased by two per stage between CS17 and CS19. The maximum branching number was 10.9 ± 1.1. Branching increased by one per CS between CS19 and CS 23. The total end-branching number was squared between the start and the end of the embryonic period. Trigeminal branches were observed occasionally and comprised 11.1% of the total amount of branching at CS17, 1.4% at CS18, 8.8% at CS19, 8.5% at CS20, 10.4% at CS21, 6.2% at CS22, and 2.8% at CS23.

**Table 1:** Development of the metanephros and the urinary collecting system during the human embryonic period.

		Length (mm)				Volume (mm3)				Ratio (v/v; %)		UCS end-branching						Number of Nephrons					
		Crown-rump		Longitudinal metanephros		UCS		Total kidney		UCS/total kidney		Average generation		Maximum generation		Total amount		Total amount		Connected to the UCS		per UCS end-branch	
CS	(n)	mean	±SD	mean	±SD	mean	±SD	mean	±SD	mean	±SD	mean	±SD	mean	±SD	mean	±SD	mean	±SD	mean	±SD	mean	±SD
14	8	6.8	0.6	0.3	0.0	0.6	0.4	5.2	1.1	9.9	6.8	0.0	0.0	0.0	0.0	1.0	0.0	0.0	0.0	0.0	0.0	0.00	0.00
15	10	8.4	1.4	0.4	0.1	1.1	0.7	10.0	2.4	10.2	5.5	0.8	0.0	0.8	0.4	1.8	0.4	0.0	0.0	0.0	0.0	0.00	0.00
16	10	8.9	0.7	0.4	0.1	1.1	0.5	12.4	1.8	8.9	4.1	1.0	0.0	1.0	0.0	2.0	0.0	0.0	0.0	0.0	0.0	0.00	0.00
17	10	11.5	1.9	0.7	0.1	0.7	0.3	22.0	5.8	3.0	0.6	1.6	0.4	1.9	0.6	3.2	0.8	0.0	0.0	0.0	0.0	0.00	0.00
18	10	13.5	1.1	0.9	0.2	1.1	0.3	42.1	18.5	2.9	0.7	3.1	0.8	4.2	1.4	8.1	3.2	0.0	0.0	0.0	0.0	0.00	0.00
19	10	15.4	2.4	1.0	0.1	2.5	0.5	109.6	27.3	2.4	0.5	4.6	1.3	6.5	0.7	19.8	5.5	4.6	4.2	0.2	0.4	0.21	0.14
20	10	18.2	1.9	1.0	0.1	4.8	1.0	177.3	38.8	2.7	0.1	5.5	1.1	7.5	0.8	42.4	7.7	27.7	13.0	3.6	4.9	0.64	0.24
21	10	19.9	2.0	1.2	0.1	7.7	2.0	318.4	95.9	2.5	0.4	6.3	1.2	8.8	0.4	74.1	15.2	61.7	27.6	21.2	14.9	0.81	0.25
22	12	22.1	0.8	1.3	0.2	15.8	4.1	525.7	226.1	3.2	0.8	7.5	1.3	9.9	0.9	124.5	34.6	135.2	61.9	57.1	41.1	1.08	0.25
23	10	26.4	1.4	1.8	0.2	90.7	72.6	1144.0	417.0	7.3	3.7	8.7	1.6	10.9	1.1	210.6	61.9	289.6	122.8	176.9	84.6	1.34	0.49

CS: Carnegie stage; SD: standard deviation; UCS: urinary collecting system

Nephrons were first observed at CS19. The total number of nephrons was 4.2 ± 4.2 at CS19 and increased to 289.6 ± 122.8 at CS23. The number of nephrons connected to the UCS was 176.9 ± 84.6 at CS23. The number of nephrons per UCS end-branching was very low but increased slightly from CS19 (0.21 ± 0.14) to CS23 (1.34 ± 0.49).

### Renal pelvis and calyx epithelium histology

**Fig 2:**
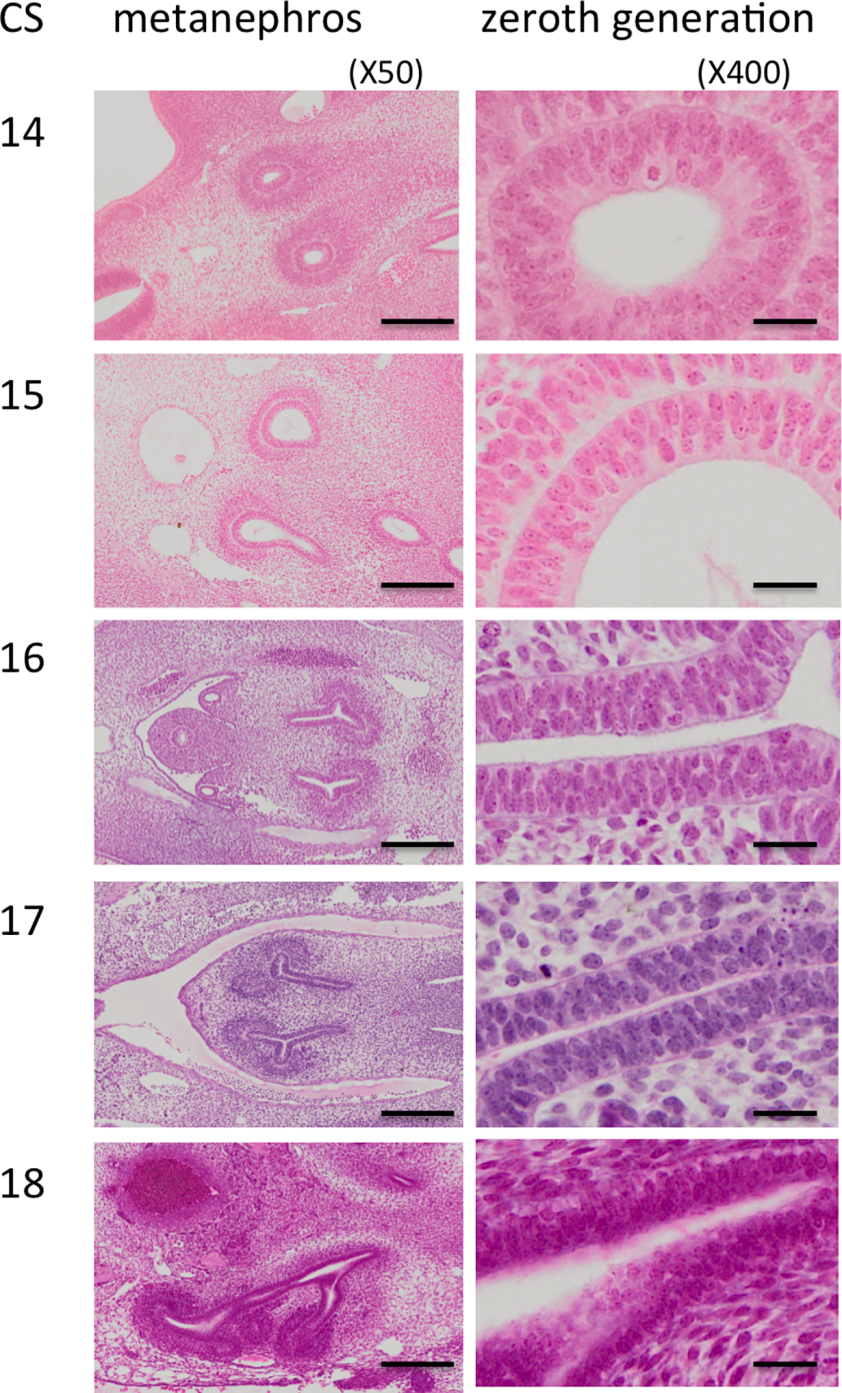
Renal pelvis epithelium (zeroth generation) between CS14 and CS23. Scale = 200 μm for x50, 20 μm for x400.

**Fig 3:**
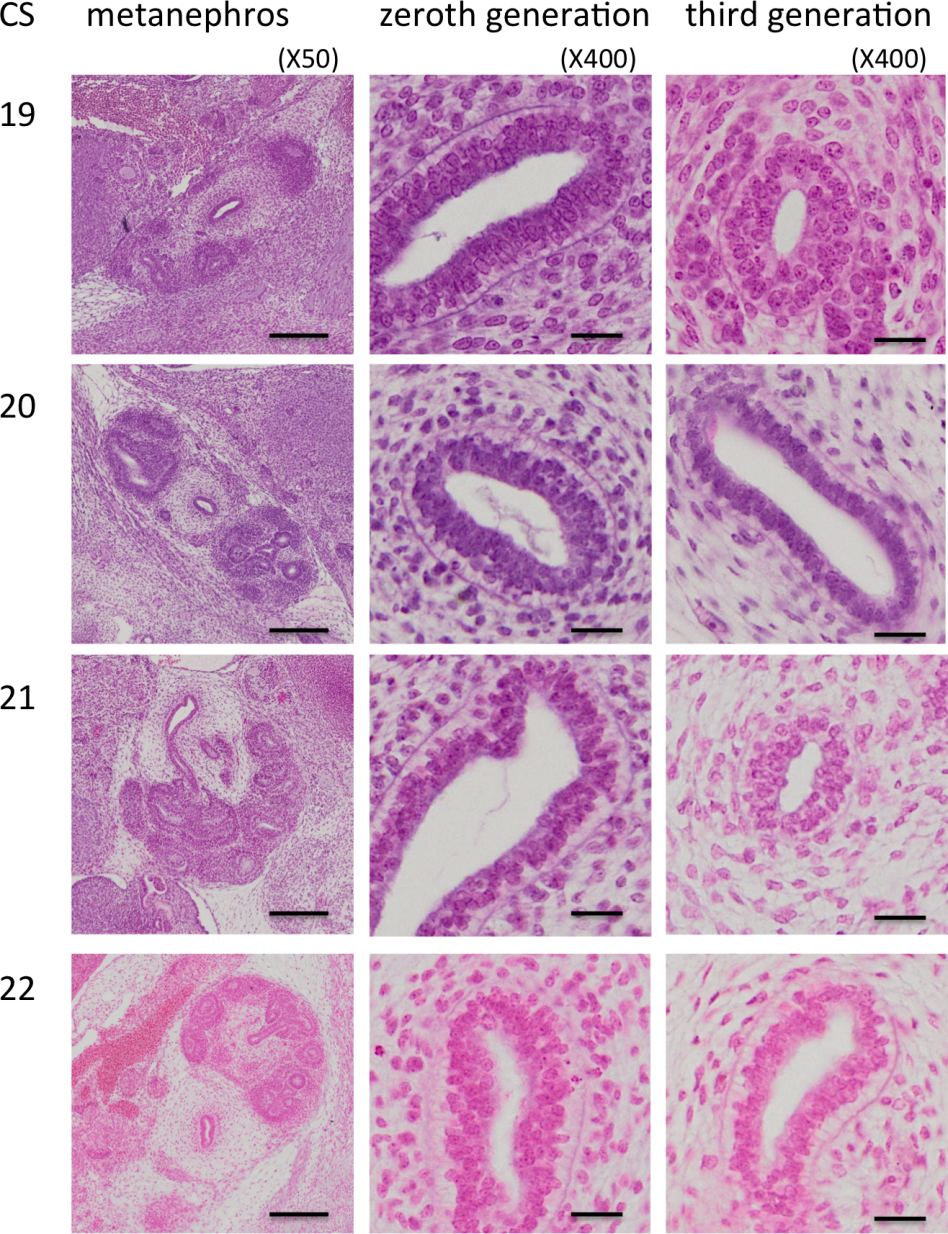
Renal pelvis histology during the embryonic period. Numbers indicate Carnegie stage. Scale = 200 μm for x50, 20 μm for x400.

The epithelium was observed between the zeroth and third UCS generations during the embryonic period. It may correspond to the renal pelvis (Figs 2 and 3). Between CS14 and CS22, it was columnar and had multilayered nuclei that moved from the basal side at CS14 and CS15 to the middle at CS16 and CS17, and then to the apical side after CS18. Surprisingly, mitotic nuclei were located on the apical side for all stages. The cytoplasm on the basal side was clear after CS19, possibly coinciding with glycogen storage. The apical border of the epithelium became thick, irregular, and opaque by CS22.

The epithelial development timeline for the third generation was essentially the same as that for the zeroth generation until CS20. In the third generation, nuclei were in the middle of the epithelium at CS19. However, they were on the apical side of the epithelium at CS20 and CS21. The apical border of the epithelium was thinner and clearer in the third generation than it was in the zeroth generation at CS22. Secreted fluid was occasionally observed in the histological sections.

### Expansion of the urinary collecting system observed at Carnegie stage 23

**Fig 4:**
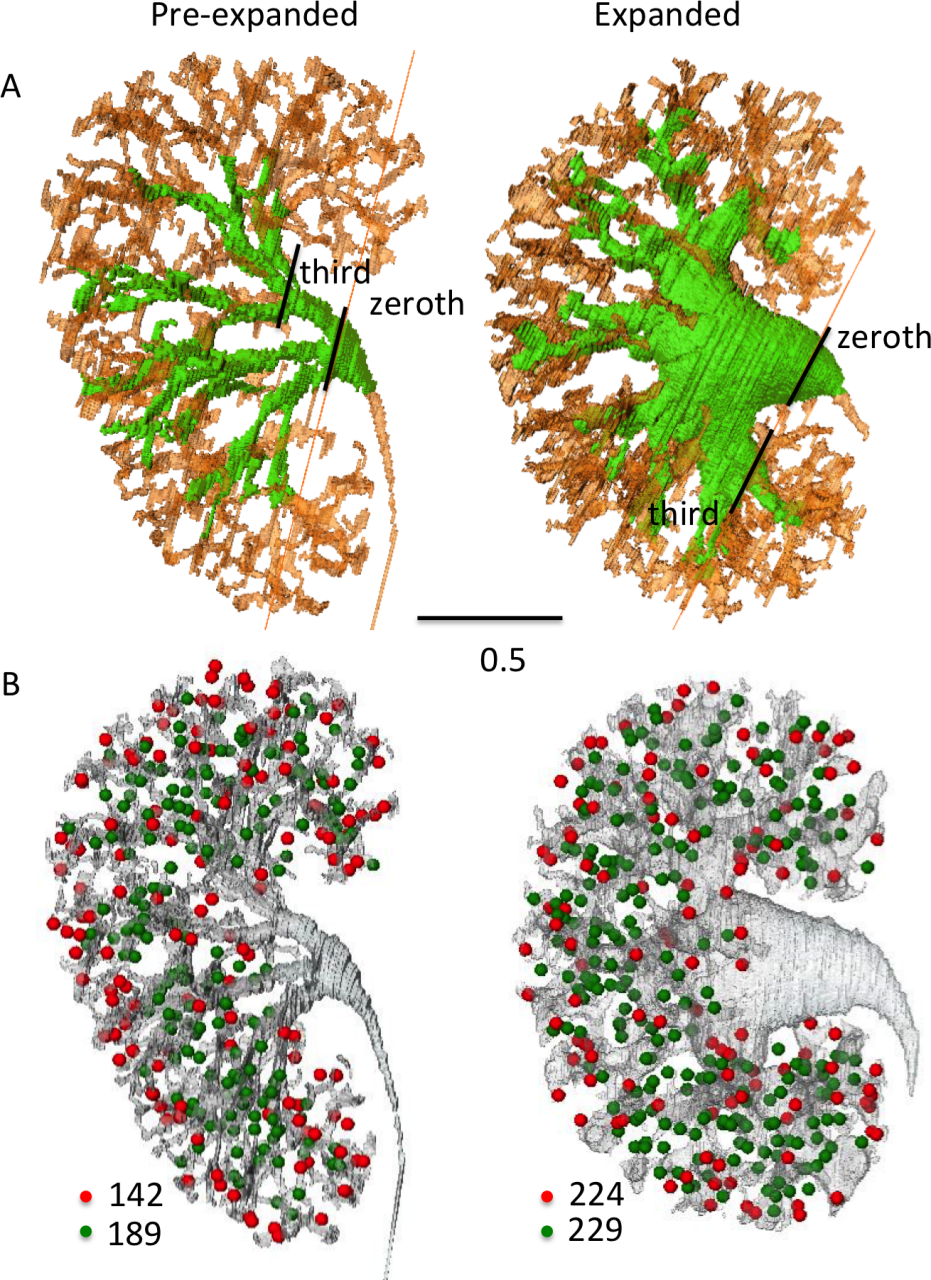
Urinary collecting systems (UCS) of the pre-expanded and expanded groups at CS23. (A) Expanded UCS are shown in light green. Histology of the zeroth (0^th^) and third (3rd) generations are indicated by lines. Scale bar = 0.5 mm. (B) Nephrons connected to UCS (green circle) and not yet connected (red circle) are shown.

Three-dimensional reconstructions of CS23 revealed metanephros with expanded renal pelvis in four of the ten samples and pre-expanded renal pelvis in the remaining six (Fig 4; S10–S13 Videos). Morphological proportions of the UCS differed among groups. We then divided the ten CS23 metanephroses into pre-expanded and expanded groups and compared their histological and morphometric features.

UCS expansion and 3-D morphological differences were subsequently observed. The UCS expanded from the zeroth to approximately the tenth generation in the samples of expanded UCS (green in Fig 4). These may correspond to the renal pelvis and the calyx in adult kidneys.

### Histology

**Fig 5:**
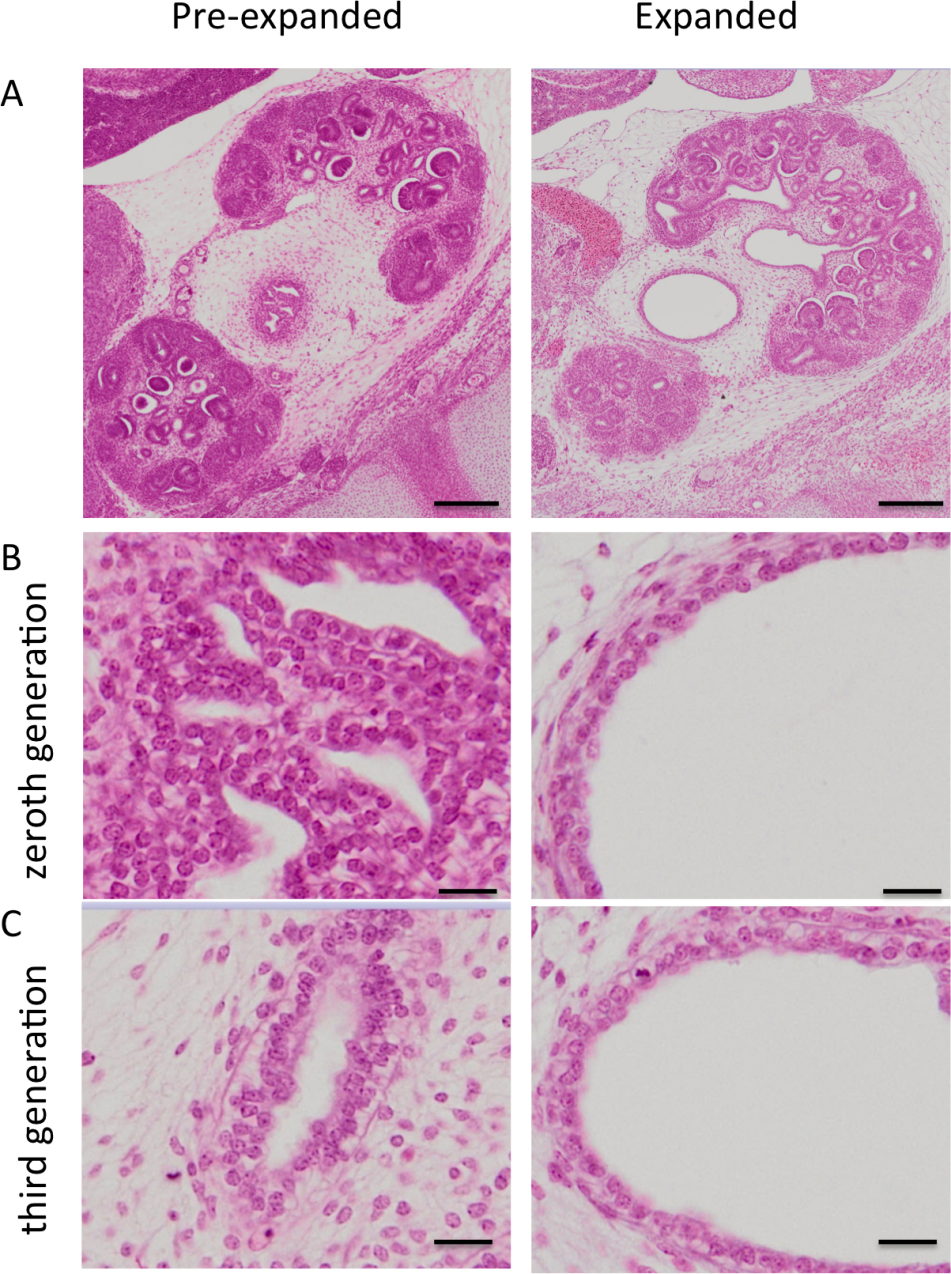
Histological sections of metanephroses stained with hematoxylin & eosin. Comparison between pre-expanded and expanded groups. A) Lower magnification (50×) showing sagittal section of whole metanephros. Scale bar = 20 μm. B) Higher magnification (400×) of renal pelvis at zeroth branching generation. Scale bar = 200 μm. C) Higher magnification (400×) of renal pelvis at third branching generation.

In the pre-expanded group, the renal pelvis epithelium was folded and stratified at the zeroth generation. In the expanded group, a single cuboidal epithelium was present (Fig 5). The renal pelvis epithelium was thinner in the expanded group at the zeroth and third generations. The apical border of the epithelium was irregular and opaque.

### Morphometry

The UCS volume in the expanded group was significantly higher than that of the pre-expanded group (171.0 ± 38.5 mm^3^ vs. 37.2 ± 4.1 mm^3^; p < 0.05). These results are consistent with the 3-D morphology described in Table 2.

**Table 2:** UCS morphometry in expanded- and pre-expanded groups at CS23.

Group	Pre-expanded group	Expanded group	*P
Sample Number (n)	6	4	
UCS volume (mm3)	37.2 ± 4.1	171.0 ± 38.5	<0.05
Crown-rump length (CRL) (mm)	25.83 ± 0.57	27.20 ± 2.12	0.39
Longitudinal metanephros length (mm)	1.67 ± 0.23	1.93 ± 0.12	0.06
Whole metanephros volume (mm3)	884.5 ± 302.8	1533.2 ± 179.5	<0.05
Volume of the whole metanephros except the UCS (mm3)	847.3 ± 305.9	1362.2 ± 141.8	<0.05
Ratio of the UCS to the metanephros (v/v; %)	4.86 ± 2.40	11.05 ± 1.22	<0.05
Total number of nephrons	222.2 ± 111.4	390.8 ± 42.9	<0.05
Number of nephrons connected to UCS	130.8 ± 80.1	246.0 ± 13.2	<0.05
Average generation of UCS end-branching	9.05 ± 1.54	8.26 ± 0.41	0.39
Maximum generation of UCS end-branching	10.8 ± 1.2	11.0 ± 1.2	0.82
Total amount of UCS end-branches	195 ± 68.2	234 ± 50.2	0.39
Number of UCS-end branches per average generation of UCS end-branching	21.0 ± 4.8	28.5 ± 7.1	0.20

CS: Carnegie stage; SD: standard deviation; UCS: urinary collecting system

The crown-rump lengths (CRL) were comparable in both groups (27.2 ± 2.12 mm in the expanded group and 25.83 ± 0.57 mm in the pre-expanded group; *P* = 0.39). The longitudinal metanephros was slightly longer in the expanded group (1.93 ± 0.12 mm) than the pre-expanded group (1.67 ± 0.23 mm); however, this difference was not statistically significant (*P* = 0.06).

The volume of the whole metanephros in the expanded group (1532.2 ± 179.5 mm^3^) was significantly higher than that of the pre-expanded group (884.5 ± 302.8 mm^3^) (*P* < 0.05). The UCS: whole metanephros ratio in the expanded group (11.05 ± 1.22%) was significantly higher than that of the pre-expanded group (4.86 ± 2.40%) (*P* < 0.05). The total number of nephrons in the expanded group (390.8 ± 42.9) was also significantly higher than that of the pre-expanded group (222.2 ± 111.4) (*P* < 0.05), and the number of nephrons connected to the UCS in the expanded group (246.0 ± 13.2) was significantly higher than that of the pre-expanded group (130.8 ± 80.1) (*P* < 0.05).

The maximum and average end-branching generations of the UCS were comparable in both groups. The maximum end-branching generations were 11.0 ± 1.2 in the expanded group and 10.8 ± 1.2 in the pre-expanded group (*P* = 0.82). The average end-branching generations were 8.26 ± 0.41 in the expanded group and 9.05 ± 1.54 in the pre-expanded group (*P* = 0.39). The total amounts of UCS end-branching were comparable in both groups (234.0 ± 50.2 in the expanded group and 195 ± 68.2 in the pre-expanded group) (*P* = 0.39). Number of UCS-end branches per average generation of UCS-end branching were comparable in both groups (28.5 ± 7.1 in the expanded group and 21.0 ± 4.8 in the pre-expanded group) (P = 0.20).

### Distribution of UCS branching

**Fig 6:**
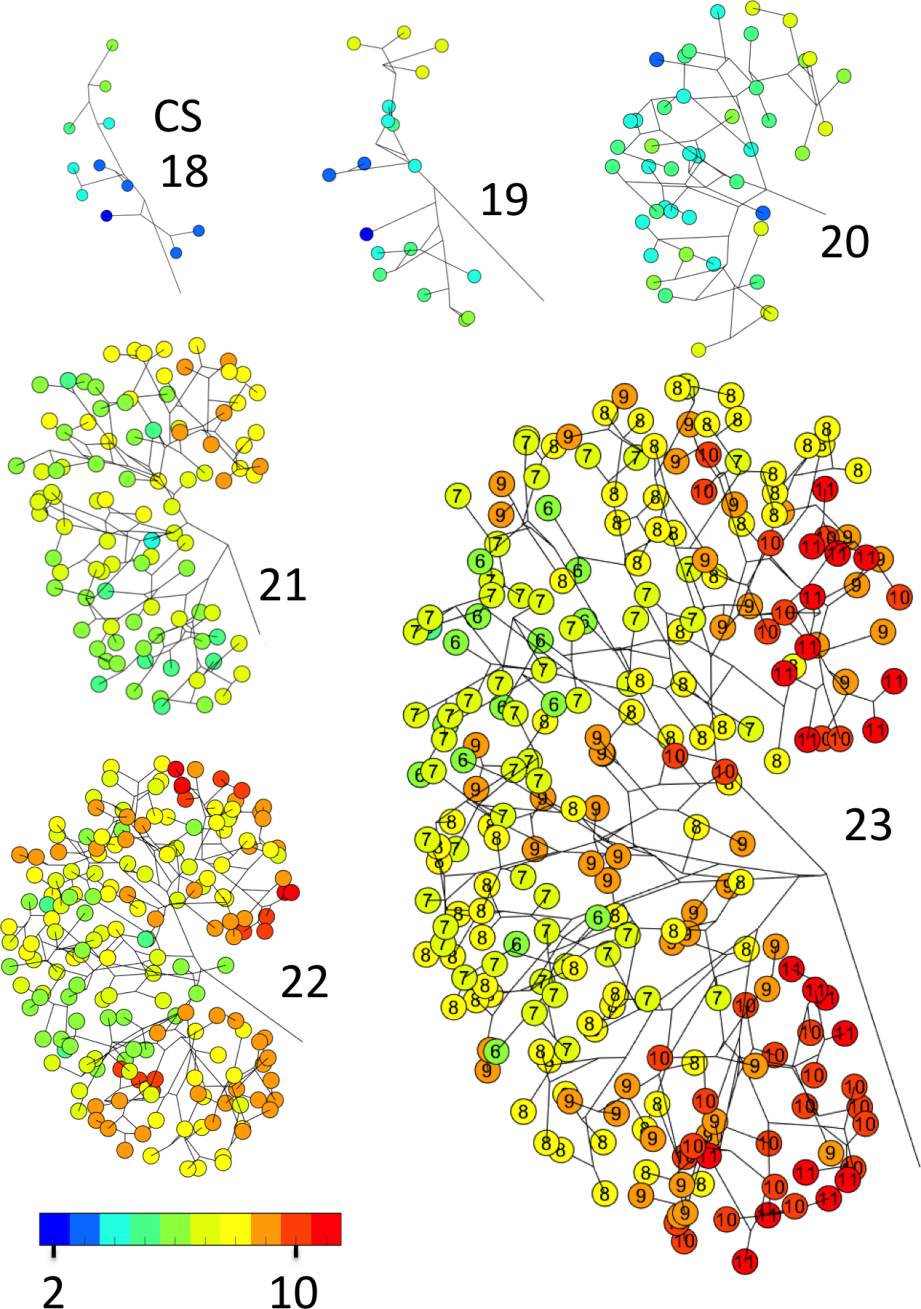
UCS branching tree showing the generation of end-branching. The generation of each end-branch is represented by a circled number. The corresponding colors are indicated by a colored bar.

The UCS rapidly developed circular sectors from the zeroth generation (renal pelvis) of the branching tree. Thus, to determine whether the rates of branching morphogenesis differed among the lobes, the generation of all end-branching was calculated and presented in the form of a UCS branching tree (Fig 6).

The UCS branching tree shows that the distribution of the generation of the end-branching differed among the four lobes. The generation of end-branching was higher in the polar than in the interpolar lobes. The distribution of the generations of end-branching among the four lobes significantly differed between CS18 and CS23 (*P* < 0.05; Wilcoxon signed-rank test) (Table 3). Distribution of the generations of end-branching between PL and IL differed significantly between CS18 and CS23 (*P* < 0.05; Mann-Whitney *U* test).

**Table 3:** Generation of end-branching according to Polar lobe Group and Interpolar lobe Group.

Carnegie stage	18		19		20		21		22		23		
	median	range	median	range	median	range	median	range	median	range	median	range	
Polar lobes	4	(2–6)	5	(4–7)	6	(5–8)	6.5	(5–8)	8	(6–9)	8	(7–10)	
Upper polar	4	(2–5)	5	(4–7)	6	(5–8)	7	(6–8)	8	(7–9)	8	(7–9)	P < 0.05
Lower polar	4	(4–6)	5	(4–6)	6	(6–7)	6	(5–8)	7.5	(6–9)	8	(7–10)	
Interpolar lobes	2	(2–3)	3	(2–4)	4	(3–5)	5	(3–7)	5	(3–8)	6.5	(2–8)	
Upper interpolar	2	(2–3)	3	(2–3)	4	(3–5)	5	(3–7)	5	(3–7)	7	(2–8)	
Lower interpolar	2	(2–3)	3.5	(2–4)	4	(4–5)	6	(5–7)	6	(4–8)	6	(6–8)	

*: calculated with the Mann–Whitney *U* test

## Discussion

The present study investigated UCS development during the embryonic period, during which bifid branching morphogenesis occurs. This study plotted the CS timeline. It comprised the 3-D structural morphogenesis of UCS and the quantification of its branching, including the total amount of end-branching, average and maximum number of generations, deviations in the metanephros, total number of nephrons and those connected to the UCS, differentiation of the urothelial epithelium in the renal pelvis, and timing of the rapid expansion of the renal pelvis.

In the early phase of morphogenesis, number of nephrons per UCS end-branching was low (0.21 ± 0.14 at CS19, 1.34 ± 0.49 at CS23), indicating that bifid branching occurred rapidly while the formation of nephrons followed. Such a tendency may be more prominent in mice [8,23], as bifid branching proceeds at a much higher speed in mice, while formation of the nephrons is slower. The maximum number of generations and total amount of end-branching (total tip number) of the UCS in E15.5 mice exceed those in humans at CS23, whereas the total number of nephrons (glomeruli) at E15.5 is equivalent to that in human embryos at CS21 [23]. In mice, approximately 40% of nephrons are formed after birth, though the final number of nephrons in adult mice is much smaller than that in humans [9], with mice having approximately 14,000 and humans possessing 100 million nephrons per kidney [7,9].

Our study showed that the eleventh generation of maximum branching occurred in CS23. If the rates of growth and branching remained constant thereafter, the UCS would reach the fifteenth branching generation soon after CS23. Oliver [6] observed the fifteenth branching generation in a 10-week-old fetus. Potter [11,16] stated that active branching occurred in the first developmental period (5–15 weeks of gestation). The results of the present study corroborate those previously reported findings and confirm that bifid branching morphogenesis may cease at ~14–15 weeks of gestation.

Irregularities in branching speed among the lobes have been described in previous studies [6,11,16]. Potter [11,16] reported that the ureteral bud divided 4 to 6 times in the polar regions and 3 to 4 times in the interpolar regions of an 18-mm embryo. In 20-mm, 8-week embryos, there are 5–6 generations of polar branches and 3–4 generations of interpolar branches. The present study showed that this imbalance continued until the end of the embryonic period. The metanephros is formed from the interaction of the UCS with the metanephric blastema—a classical example of an epithelial-mesenchymal interaction [3]. The branching rate, then, reflects the orientation of growth required to form the metanephros. Specifically, the metanephros grows faster towards the upper and lower poles than it does towards the interpolar regions. This imbalance among the lobes may explain the fact that the metanephros elongates first in the cranial-caudal direction, forming a prominent characteristic ‘bean’ shape of the kidney rather than a fan shape. The imbalance among the lobes is also prominent in mice [8–10]. Sampogna et al [8] showed that the imbalance in branching rates among the lobes (higher rates in upper and lower poles) was prominent in mice after E13.5, reflecting the progressive asymmetric and irregular kidney shape.

A previous study stated that in an 11-week, 50-mm fetus, the UCS had a primitive renal pelvis because of the expansion of the third- to fifth generation and early calyx formation [7]. This developmental phase may correspond to the post-embryonic period. Our data revealed that the renal pelvis expands in CS23, which is earlier than that reported in a previous study. Similar findings have also reported recently [24]. Such acute dilation of the UCS has also been recognized at E15.5 (Limb Stage 12) mice [9,10].

Previous studies suggest that UCS fusion may accompany renal pelvis and calyx expansion [6,11,12]. The first 4–5 polar and 2–3 interpolar branches coalesce to form 2–4 major calyces extending from the renal pelvis. There should also be a decrease in the number of branch generations in the metanephros. Lindström et al [24] provided data, which suggests that the initiation of renal pelvis development was evident, with the early branches of the UCS partially engulfing the pelvis. They speculated that such findings point to the ongoing remodeling of the UCS. Branch resorption during formation of the renal pelvis is a recognized and experimentally supported feature of mouse kidney development [9]. However, in the present study, the average and maximum generation of UCS end-branching and the total amount of UCS end-branching did not differ significantly between the pre-expanded and expanded groups. Short et al (2014) demonstrated a shift in the relationship between the tip number and generation of UCS end-branching in mice between E15.5 and E16.5, and indicate that branch resorption and/or fusion occurred at those phases for mice [9]. However, in our present study, the number of UCS end-branches per average generation of UCS end-branching were comparable in both groups. Thus, our present data do not indicate that branch fusion and/or resorption occurs during the expansion process in CS23. The expansion mechanisms at this stage have not yet been elucidated.

The CS17 mesonephros has epithelial plaques in the visceral layer of the glomerular capsule and it can, as a result, produce urine [25]. The cloacal membrane ruptures under urinary pressure [26] at CS18 or CS19 [19]. A similar mechanism might contribute to this rapid expansion. Namely, glomerular filtrate may increase UCS pressure, forcing it to expand rapidly. Potter also presented the hypothesis that urinary secretion contributes to the expansion of the pelvis and calyces [16]. We detected the nephrons since CS19 and found that the total numbers of nephrons and of those connected to the UCS increased until CS23. Moreover, the total numbers of nephrons connected to the UCS was significantly higher in the expanded group than in the pre-expanded group. At these stages, the nephrons connected to the UCS produce and secrete fluid into the UCS. Previous studies have suggested that the human metanephros starts to contribute to amniotic fluid volume at CS23 or earlier [27]. Lindström et al [24] used immunofluorescent analysis with several differentiation markers to demonstrate that glomerular filtration may be occurring at CS23. Further studies will be necessary to substantiate this hypothesis.

Urothelial epithelium maturation in the renal pelvis is required before urine production, which enables the UCS to expand at CS23. In the present study, the epithelium differed between the pre-expanded- and expanded groups at the zeroth generation. The urothelial epithelium was folded and stratified in the renal pelvis of the pre-expanded group, whereas a single cuboidal epithelium was present in the expanded group. The epithelium was thinner in the expanded group than it was in the pre-expanded group. Therefore, at CS23, the urothelial epithelium may be differentiated to allow expansion in the renal pelvis up to approximately the tenth generation. The unexpanded regions in the periphery of the UCS may not have urothelial epithelium and cannot expand at CS23. To identify the generations at which the collecting ducts differentiate from the UCS, it will be necessary to localize the border between the urothelial- and the cuboidal epithelia. Imunohistochemistry using molecular markers of differentiation of the urothelium, such as uroplakin, may be necessary to define the status of these cell populations.

During the late branching phase in mice (after E15.5), global remodeling and sculpting of the tree occurs, leading to the formation of the renal pelvis [8, 9, 28]. This includes simultaneous proliferation and apoptosis for the remodeling of initial branch segments and resorption of several segments into the expanding renal pelvis. Differentiation of the urothelium and production of urine are also observed at E15.5 [29]. In humans, renal pelvis expansion at CS 23 observed in the present study may be the first step, and the remodeling that occurs to form the medullary collection duct arrays (renal papilla and calyx) may occur in the next step. It remains unknown whether such remodeling in mice is consistent with that which may be observed during later stages of development in humans. Information regarding the precise mechanism of the remodeling process in humans is still lacking.

The present study followed UCS development during the embryonic period, which was found to mainly consist of bifid branching morphogenesis. The CS timeline we plotted may be a useful and accurate indicator of UCS development. Failure or delay of rapid expansion might be detected in the form of abnormal development of the glomerular capsule and/or the UCS, including the urothelial epithelium. It may also serve to forecast functional renal capacity after differentiation and growth.

## Supporting information

S1 Video Metanephros at CS14 (ID_14540). (AVI)

S2 Video Metanephros at CS15 (ID_10304). (AVI)

S3 Video Metanephros at CS16 (ID_5709). (AVI)

S4 Video Metanephros at CS17 (ID_33763). (AVI)

S5 Video Metanephros at CS18 (ID_24992). (AVI)

S6 Video Metanephros at CS19 (ID_16696). (AVI)

S7 Video Metanephros at CS20 (ID_7271). (AVI)

S8 Video Metanephros at CS21 (ID_41). (AVI)

S9 Video Metanephros at CS22 (ID_5214). (AVI)

S10 Video Metanephros with pre-expanded renal pelvis at CS23 (ID_4381). (AVI)

S11 Video Metanephros with expanded renal pelvis at CS23 (ID_12481). (AVI)

S12 Video Metanephros with pre-expanded renal pelvis and nephrons at CS23 (ID_4381). (MOV)

S13 Video Metanephros with expanded renal pelvis and nephrons at CS23 (ID_12481). (MOV)
